# Testing Effects on Shear Transformation Zone Size of Metallic Glassy Films Under Nanoindentation

**DOI:** 10.3390/mi9120636

**Published:** 2018-11-30

**Authors:** Yi Ma, Yuxuan Song, Xianwei Huang, Zhongli Chen, Taihua Zhang

**Affiliations:** 1 College of Mechanical Engineering, Zhejiang University of Technology, Hangzhou 310014, China; 1111702002@zjut.edu.cn (Y.S.); huangxw@zjut.edu.cn (X.H.); 2 China Jiliang University, Hangzhou 310018, China; chenzhongli@cjlu.edu.cn; 3 Institute of Solid Mechanics, Beihang University, Beijing 100191, China

**Keywords:** metallic glass, nanoindentation, creep, strain rate sensitivity, shear transformation zone

## Abstract

Room-temperature creep tests are performed at the plastic regions of two different metallic glassy films under Berkovich nanoindetation. Relying on the strain rate sensitivity of the steady-state creep curve, shear transformation zone (STZ) size is estimated based on the cooperative shear model (CSM). By applying various indentation depths, loading rates, and holding times, the testing effects on the STZ size of metallic glasses are systematically studied. Experimental results indicate that STZ size is greatly increased with increased indentation depth and shortened holding time. Meanwhile, STZ size is weakly dependent on the loading history. Both the intrinsic and extrinsic reasons are discussed, to reveal the testing effects on the nanoindentation creep flow and STZ size.

## 1. Introduction

Metallic glasses are at the cutting edge of new-structure material research and have great potential to be utilized as engineering materials for their excellent mechanical properties [1,2,3,4]. Due to its unique atomic configuration, i.e., non-crystalline but with a short-range order structure, metallic glass is also an important part of condensed matter physics. However, localized shear banding dominates in plastic deformation and catastrophic failure always occurs under tension [5]. A great deal of research efforts have focused on revealing the mechanism of plastic deformation and improving workability in metallic glasses. Owing to the original work of Argon [6], the deformation unit with a local rearrangement of atoms, also referred to as the shear transformation zone (STZ), has been widely applied to analyze the low-temperature deformation of metallic glasses. Being different from a structural defect, STZ is defined by its transience, i.e., it can only be identified from the atomic structures before and after deformation. The details of STZ evolution are mostly studied using computer simulations, in relation to its shape, configuration, and activation mechanism [7,8,9,10].

In recent years, following the cooperative shear model (CSM) by Johnson and Samwer [11], Pan et al. developed an experimental method to estimate STZ volume relying on strain rate sensitivity (SRS) through rate-jump nanoindentation [12,13,14]. In Pan’s work, STZ size displayed a strong correlation with Poisson’s ratio and structure state [12,15], and could be closely tied to the ductility of bulk metallic glasses. However, the experimental results determined using the rate-jump method are in doubt. Bhattacharyya et al. revealed that pile-up in nanoindentation would significantly affect the SRS values using the rate-jump method [16], hence the questionable STZ volume. Moreover, strain rate sensitivities in some kinds of metallic glasses are extremely low, and even negative. Thus, the measuring error would be significant for calculating SRS values and the corresponding result concerning STZ size might be unreliable.

Indentation creep has been the most extended method for studying strain rate sensitivity [17]. The holding stage during nanoindentation could be much more time-saving in comparison to the traditional creep test, due to its high accuracy for recording creep displacement [18,19]. Nanoindentation creep investigations have been widely performed in small-scale nanocrystalline materials [20,21,22,23]. In the authors’ previous work, spherical nanoindentation creep behaviors in metallic glassy films with various compositions were carefully studied [24]. Furthermore, STZ sizes and their correlations with intrinsic parameters of metallic glasses such as sample dimension [25,26], structure state [27,28], residual stress [29], and glass transition temperature (*T*_g_) [24], were obtained, relying on the SRS of creep curves. It has been recognized that nanoindentation creep behaviors are dependent on testing conditions in both crystalline and amorphous alloys [30,31,32,33,34,35,36]. Now, one question naturally arises: Does the STZ size of metallic glass also depend on extrinsic testing effects? With this in mind, two distinct metallic glassy films are prepared, which could provide a clean and smooth surface for nanoindentation. In the present study, the testing effects of indentation depth, loading rate, and holding time on STZ size are studied using the creep method.

## 2. Materials and Methods

Zr-Cu-Ni-Al and La-Co-Al films were deposited on a silicon wafer in a DC magnetron sputtering system at room temperature in pure argon gas. The 2-inch target alloys adopted in the chamber were Zr_64_Cu_16_Ni_10_Al_10_ and La_60_Co_20_Al_20_, at.%, which was prepared from high-purity (99.99 wt.%) elements using vacuum casting. The target was installed at the bottom while the silicon wafer was stuck onto the sample platform, which was right above the target. The target-to-substrate distance was kept constant, equal to 100 mm. The base pressure of the chamber was kept at about 5 × 10^−7^ Torr before deposition and the working argon pressure was set at about 1 mTorr. The power on the target was fixed at 120 W during the deposition and the sputtering time was two hours. The film thickness could be directly measured from the cross-section using a scanning electron micrograph (SEM). By means of an X-ray energy dispersive spectrometer (EDS) attached to the SEM, the chemical compositions of as-prepared films were detected as Zr_55_Cu_15_Ni_13_Al_17_ and La_55_Co_20_Al_25_, respectively. The amorphous nature was confirmed using X-ray diffraction (XRD) with Cu K_α_ radiation [24].

Nanoindentation experiments were conducted at a constant temperature of 20 °C on an Agilent Nano Indenter G200 with the dynamic contact module (DCM), by which higher resolution in both force and displacement and less sensitivity to the environment could be attained. The constant temperature was controlled by the air condition. A standard Berkovich indenter was applied, the tip of which was detected to be perfect using transmission electron microscopy (TEM), to avoid the tip bluntness effect on the mechanical response [37,38]. The constant load-holding method was used in this study to explore the creep flows and strain rate sensitivities under various testing conditions. The indenter was held for 500 s at various depths of 50 nm, 100 nm, 200 nm, and 350 nm (only for La-Co-Al film), with a constant loading rate of 0.2 mN/s. At a constant holding depth of 200 nm, the loading rate effect on the creep flow was studied; during these tests, four different loading rates of 0.035 mN/s, 0.075 mN/s, 0.2 mN/s, and 0.75 mN/s were employed. Furthermore, five different holding durations of 15 s, 50 s, 100 s, 500 s, and 1000 s were adopted to study the holding time effect on the value of strain rate sensitivity and STZ size. All the above-mentioned testing parameters were carefully chosen, in view of the potential disturbances of the substrate effect, tip bluntness effect, and the influences of thermal drift and instrument error on experimental results. The creep tests were launched until thermal drift was reduced to below 0.02 nm/s. Meanwhile, drift correction, which was calibrated at 10% of the maximum load during the unloading process, was strictly performed. To ensure the reliability of the creep results, twenty nanoindentation measurements were conducted for each test.

## 3. Results and Discussion

**Figure 1:**
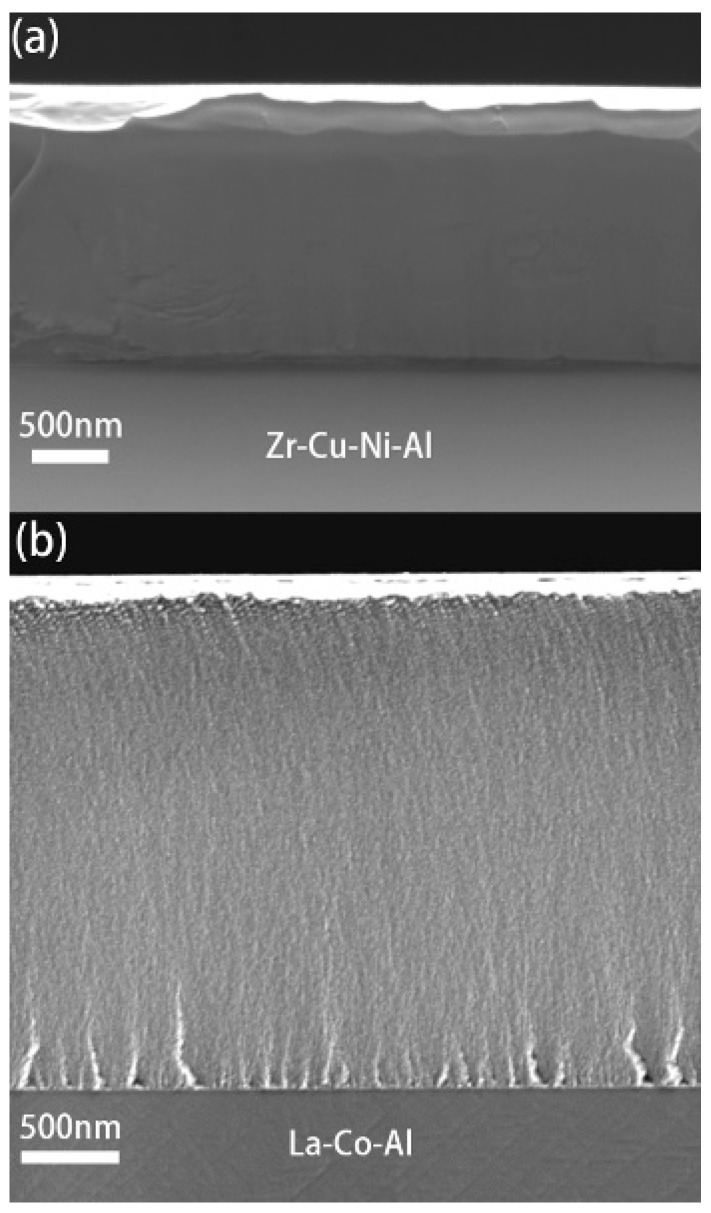
Typical cross-sections of: (**a**) Zr-Cu-Ni-Al; and (**b**) La-Co-Al films using scanning electron micrograph (SEM), film thickness could be measured.

**Figure 2:**
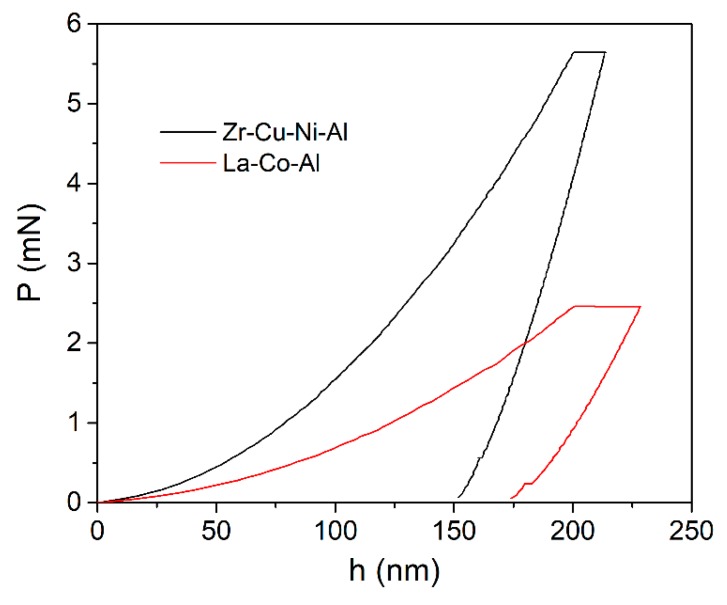
Representative load-displacement curves of the 500-s holding creep tests with a maximum displacement of 200 nm for Zr-Cu-Ni-Al and La-Co-Al films.

**Figure 3:**
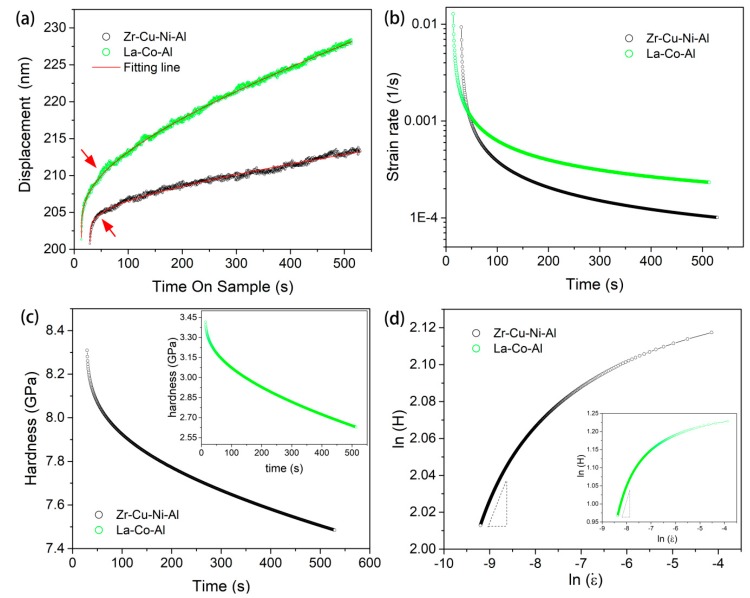
(**a**) the creep displacements during the holding stage versus holding time, which can be perfectly fitted using an empirical law, and the critical point of creep transition from transient stage to steady-state stage is marked with an arrow; (**b**) the creep strain rate versus holding time; (**c**) the hardness versus holding time; (**d**) the log-log correlation between the hardness and strain rate obtained from the creep, strain rate sensitivity can be thus estimated by linear fitting of the steady-state part.

Figure 1 shows the film cross-sections of both samples; the thicknesses of Cu-Zr-Ni-Al and La-Co-Al films could be directly measured as 1.6 μm and 2.7 μm, respectively. Figure 2 shows the typical load-displacement (*P*-*h*) curves of 500-s creep tests with a holding depth of 200 nm and a loading rate of 0.2 mN/s. The room-temperature creep deformation could be clearly observed at the holding stage. At the onset of the holding stage, severe plastic deformation beneath the Berkovich indenter had already occurred. This was, intrinsically, why creep deformation could easily occur even in many high-melting point materials under nanoindentation at room temperature. The creep flows of metallic glassy films could be more intuitively recognized from the relationship between the creep displacements and holding time, as plotted in Figure 3a. The creep curves could be divided into two distinct stages, as transient creep and steady-state creep. The approximate critical point for the creep transition is marked with an arrow in Figure 3a for both samples. In comparison, the transient creep stage was much shorter in La-Co-Al than in Zr-Cu-Ni-Al. At the transient stage, the creep displacement increased relatively fast, but the creep rate dropped rapidly. Then, the creep displacement turned out to be slow and increased almost linearly with time at the steady-state stage. Clearly, creep deformation is more pronounced in La-Co-Al than in Zr-Cu-Ni-Al, which could be expected due to the lower glass transition temperature (*T*_g_) and hardness. The creep curves for both samples can be perfectly fitted (R^2^ > 0.99) using an empirical law [39]:

*h*(*t*) = *h*_0_ + *a*(*t* − *t*_0_)*^b^* + *kt* (1)

where *h*_0_ and *t*_0_ are the displacement and time at the beginning of the holding stage, and *a*, *b*, *k* are the fitting constants.

The value of the SRS exponent *m* can be evaluated via [40]:

(2) $$m=\frac{\partial \ln H}{\partial \ln\dot{\varepsilon }}$$

In the present Berkovich nanoindentation process, the strain rate during the holding stage can be calculated as $\dot{\varepsilon }=\frac{1}{h_{p}}\frac{dh_{p}}{dt}$ and the hardness is defined as $H=\frac{P}{Ch_{p}^{2}}$. *C* is the tip area coefficient and is rectified upon testing on the standard fused silica, equal to 23.6 here. $h_{p}$ is the contact depth and could be estimated as *h_p_* = *h_i_* − *εP*/*S*, where *h_i_* is the total nanoindentation displacement, *ε* = 0.72 for a Berkovich tip, and *S* is the stiffness deduced from the unloading curve. Based on the fitting line of the creep curve in Figure 3a, the variations in the strain rate and hardness as a function of the holding time are obtained, as shown in Figure 3b,c, respectively. Figure 3d shows the log-log correlation between the hardness and strain rate during the holding stage. Accordingly, *m* can be obtained by linearly fitting the part of the steady-state creep (here, we adopted the last 200-s holding part). For reliability, nine effective creep curves were employed to reach an average value of SRS for each sample.

The STZ volume can then be estimated accordingly using the cooperative shear model (CSM) of Johnson and Samwer [11], based on the SRS obtained from the creep. In the CSM model, the activation energy of the STZ is defined as:

(3) $$W_{STZ}=4R_{0}G_{0}\gamma_{C}^{2}{\left(1-\tau /\tau_{0}\right)}^{\frac{3}{2}}\xi \Omega$$

Thus, the correlation between the STZ volume Ω and the activation volume V* can be obtained through directly differentiation of the activation energy $W_{STZ}$, given by:

(4) $$\Omega =\frac{\tau_{0}}{6R_{0}G_{0}\gamma_{C}^{2}{\left(1-\tau /\tau_{0}\right)}^{\frac{1}{2}}\xi }V^{*}$$

where *R*_0_ ≈ 1/4 and ξ ≈ 3 are constants, *τ* and *τ*_0_ are threshold shear resistances at temperature *T* and 0 K, *G*_0_ is the shear modulus at 0 K, the average elastic limit *γ_C_* ≈ 0.027, *τ*_0_/*G* = 0.036, and the value of *τ*/*τ*_0_ can be estimated using the constitutive equation:

(5) τ/G=γC0−γC1(TTg)2/3

where $\gamma_{C0}=0.036\pm 0.002$, $\gamma_{C1}=0.016\pm 0.002$, the shear modulus $G\approx \frac{E}{2\left(1+\nu \right)}$ has a weak temperature dependency for a metallic glass [11]. The STZ activation volume V* can be expressed as [40]:

(6) $$V^{*}=\frac{kT}{m\tau_{y}}$$

Here, *τ_y_* is the critical shear stress upon the traditional tensile or compressive tests, with an empirical correlation of *τ_y_* ≈ *H*/3$\sqrt{3}$. The hardness value at the initial holding stage was adopted to estimate the flow stress. Once the STZ activation volume V* had been determined, STZ activation energy and volume could be calculated using Formulas (3) and (4). According to the dense-packing hard-sphere model of metallic glass [41], wherein the average atomic radius $r\approx {\left(\overset{n}{\underset{i}{\sum }}A_{i}r_{i}^{3}\right)}^{1/3}$, $A_{i}$ and $r_{i}$ are the atomic fraction and atomic radius of each element, respectively, the atoms contained in an STZ could be estimated.

### 3.1. Indentation Size Effect

**Figure 4:**
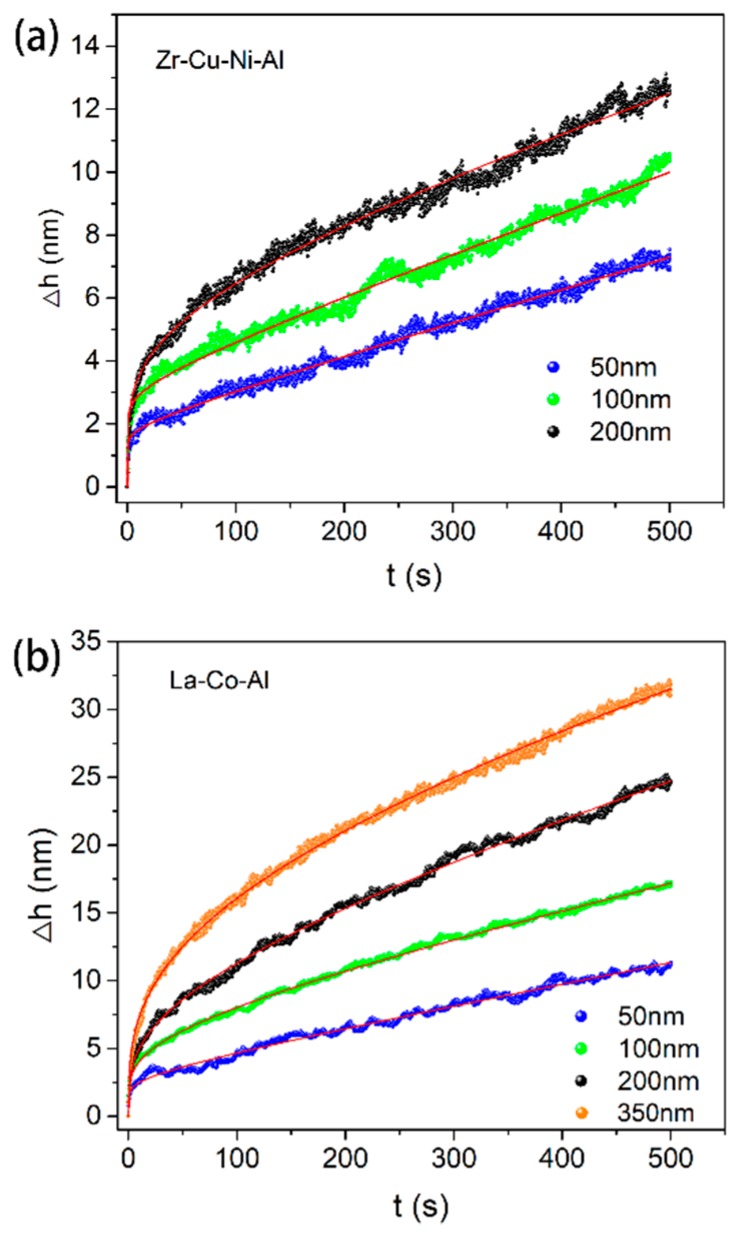
Typical creep displacements versus holding time at various initial holding depths for (**a**) Zr-Cu-Ni-Al and (**b**) La-Co-Al.

**Figure 5:**
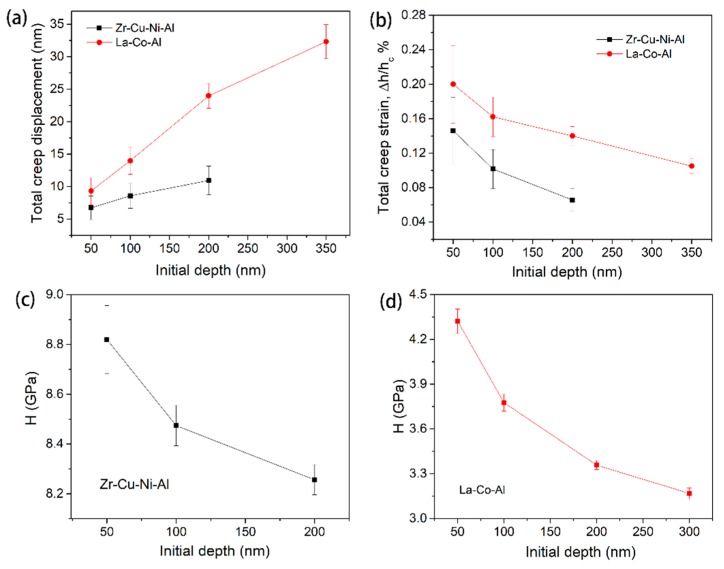
(**a**) The total creep displacement and (**b**) creep strain for at various initial holding depths for both samples; (**c**) hardness at the onset of the holding stage as a function of initial holding depth for (**c**) Zr-Cu-Ni-Al and (**d**) La-Co-Al.

Figure 4a,b shows the typical creep flows at various holding depths for Zr-Cu-Ni-Al and La-Co-Al, respectively. It should be noted that a load-holding test was not conducted at 350 nm for the Zr-Cu-Ni-Al film due to the potential substrate effect [42,43,44]. In order to recognize the indentation size effect on creep behavior more directly, the starting points (including both the holding time and creep displacement) for all the creep curves were set to be zero. Clearly, the creep flow was enhanced by increasing the initial holding depth. Furthermore, all the creep curves could be perfectly fitted using Equation (1). The total creep displacement at each holding depth was recorded for both samples, as shown in Figure 5a. The enhancement of the creep displacement by increasing the holding depth was more evident in La-Co-Al than in Zr-Cu-Ni-Al. From the perspective of structure agitation under nanoindentation, it is qualitatively claimed that the free volume content of metallic glass would be increased with pressed depth, hence promoting creep flow at a larger holding depth. In addition, the shear band density and excess free volume generated during nanoindentation would be composition-dependent in metallic glasses [45], which could be the reason for the different performances in relation to the indentation size effect in Zr-Cu-Ni-Al and La-Co-Al. Such indentation size effect on creep deformation is commonly observed in nanoindentation creep, though its mechanism is complicated. For a spherical indenter, the plastic deformation beneath the indenter would be more severe as the pressed depth increases. More excess free volume would be generated in the plastic zone, causing improved atomic mobility. It could be conceivable that a larger holding depth would facilitate creep flow (creep strain) under spherical nanoindentation, while for a standard Berkovich indenter, the imposed plastic strain and stress distribution during nanoindentation are self-similar at various pressed depths. Theoretically, creep displacement would be proportional to the initial holding depth and creep strain would be invariable under Berkovich indentation. Practically, the stress distribution beneath the indenter is much more complicated than that in uniaxial testing and creep deformation could not occur uniformly around the plastic region. Here, we define creep strain as Δ*h*/*h*_c_, where Δ*h* is the total creep displacement and *h*_c_ is the contact displacement at the beginning of the holding stage. The correlations between creep strain and initial holding depth are shown in Figure 5b for both samples. The creep strain was gradually decreased with the increased initial holding depth. This phenomenon has been revealed in crystalline/amorphous nanolaminates, where the apparatus error of “indenter overshoot” plays an important role at the very beginning of the holding stage [46]. Figure 5c,d exhibits the hardness values at the beginning of the holding stage, which apparently decrease with increasing pressed depth.

**Figure 6:**
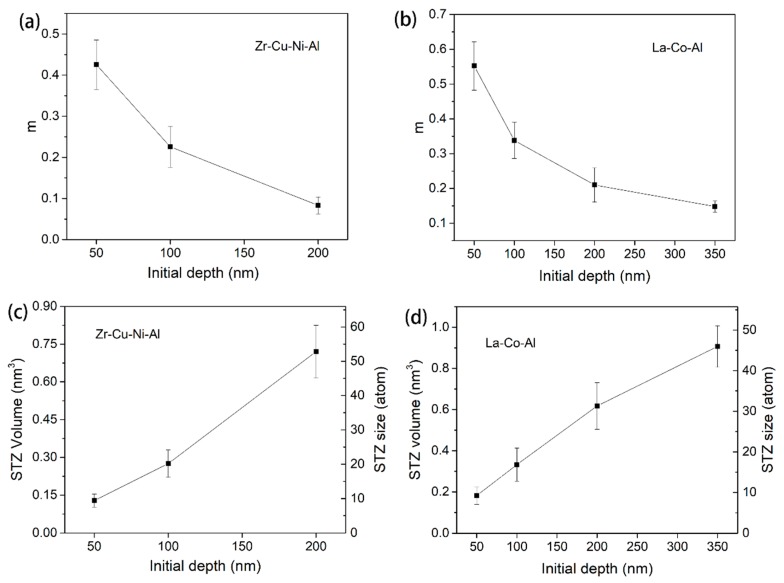
(**a**,**b**) Strain rate sensitivity, and (**c**,**d**) shear transformation zone (STZ) size as a function of initial holding depth for Zr-Cu-Ni-Al and La-Co-Al.

The SRS at various holding depths are computed using steady-state creep curves for both samples. The obtained values are in the order of 10^−1^, which is remarkably higher than those reported using the rate-jump method. The rate-jump method is conducted under quasi-static loading and suffered instantaneous plastic deformation, the results of which are always a magnitude less than those using creep. Figure 6a,b clearly shows that SRS decreased with the increasing holding depth. Based on the above values of SRS and hardness, the STZ volume and size of both samples could be determined. As depicted in Figure 6c,d, we can conclude that STZ size increases with increasing holding depth. The calculated values of STZ size increased from 10 atoms to 53 atoms for Zr-Cu-Ni-Al, and from 9 atoms to 46 atoms for La-Co-Al, as the holding depths increased from 50 nm to 200 nm and 50 nm to 350 nm, respectively. The STZ volume and size were, respectively, in the range of 0.1~1 nm^3^ and 10~60 atoms, which corresponds well with previous studies on metallic glasses detected using experimental methods and molecular dynamic simulation [47,48,49]. On the other hand, the STZ size of La-Co-Al is smaller than Zr-Cu-Ni-Al at the same holding depth, which could be due to its lower *T*_g_~500 K (730 K for Zr-Cu-Ni-Al) [50]. The glass transition temperature is a vital thermodynamic parameter of metallic glass, which is closely connected to the atomic structure and plastic deformation [51].

The indentation size effect on creep deformation and strain rate sensitivity (or stress exponent) has been widely reported in both crystalline and amorphous alloys [30,31,32,33]. However, the correlation between the activation volume of the plastic unit and pressed depth is rarely investigated. In Jang et al.’s work [49], it was reported that the STZ size of a Zr-based metallic glass was unchanged under spherical indenters with different radius using the statistical method. However, the initial conditions such as stress distribution and plastic deformation in their work were changed with different indenters, which significantly influenced the emergence of the first pop-in event. Strictly speaking, this was merely the indenter radius effect rather than the indentation size effect on STZ size. What is more, the indentation size effect on STZ size cannot be fully studied using the statistical method or rate-jump method in nanoindentation, due to their testing principles. To the authors’ best knowledge, the results presented here are the first report revealing the indentation size-dependent STZ size in metallic glasses.

### 3.2. Loading Rate Effect

**Figure 7:**
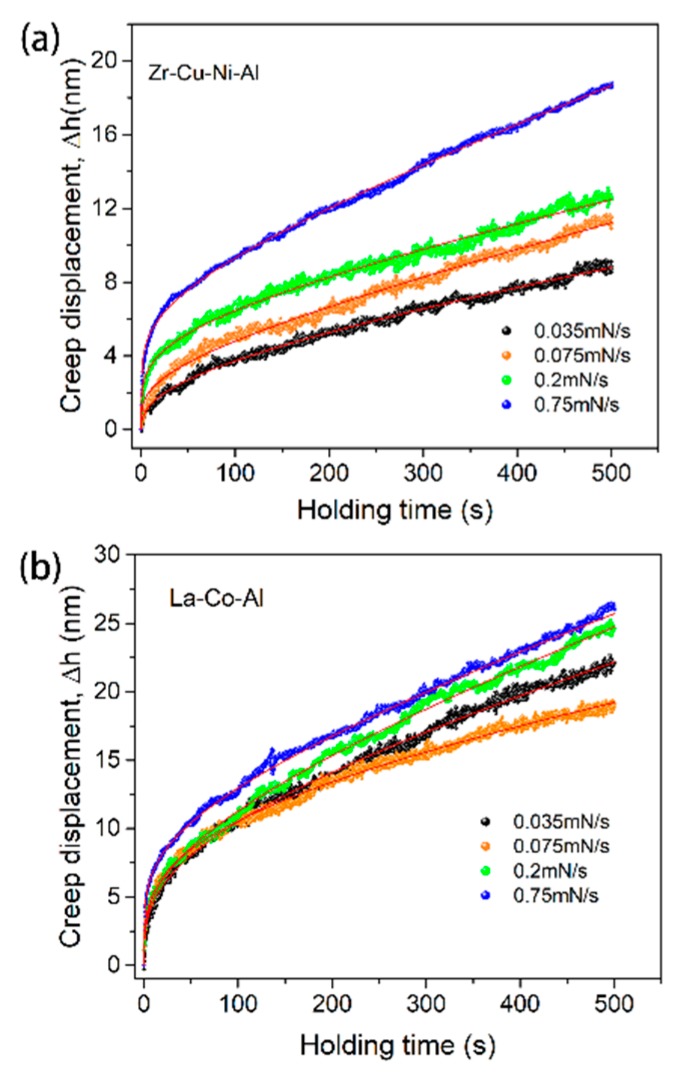
Typical creep displacements versus holding time with four different loading rates at the initial holding depth of 200 nm for (**a**) Zr-Cu-Ni-Al and (**b**) La-Co-Al; the creep curves could be perfectly fitted.

**Figure 8:**
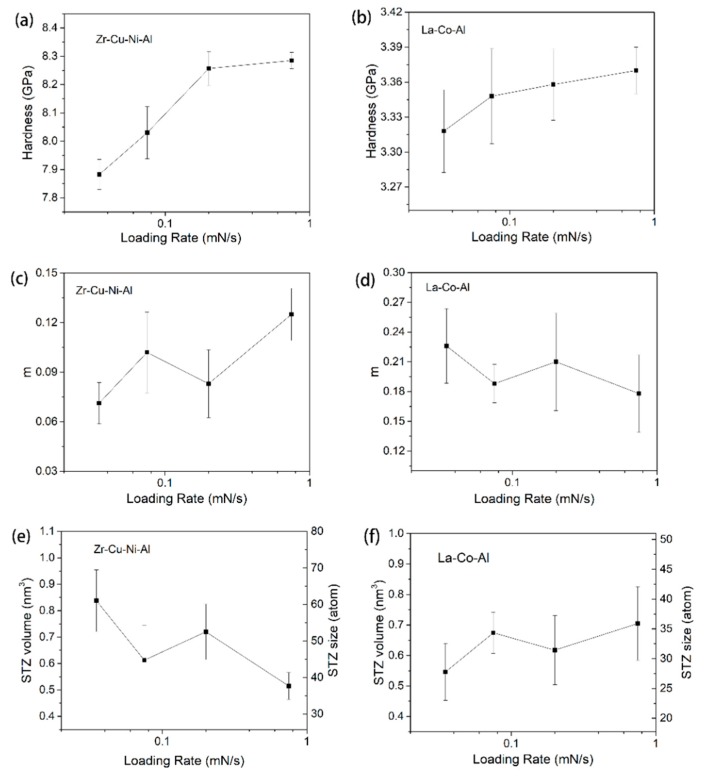
Hardness at the onset of the holding stage, strain rate sensitivity, and STZ size as a function of the loading rate for (**a**,**c**,**e**) Zr-Cu-Ni-Al and (**b**,**d**,**f**) La-Co-Al.

Figure 7 shows the typical creep curves during the holding stage under four different loading rates. The initial holding depth is constant as 200 nm for both samples. A fast loading sequence is thought to facilitate nanoindentation creep deformation during the holding stage [34,35,36]. However, the loading rate effect presented here on the creep flow of metallic glass is composition-dependent. For the Zr-Cu-Ni-Al film in Figure 7a, the creep deformation increases weakly when increasing the loading rate from 0.035 mN/s to 0.2 mN/s, and proves much more pronounced under 0.75 mN/s. Qualitatively, the stimulate effect on creep deformation by a high loading rate is more remarkable at the transient stage (0~50 s) than the steady-state stage for the Zr-Cu-Ni-Al film. For the La-Co-Al film in Figure 7b, on the other hand, the creep flows are observed to be history-independent. There is no obvious distinction among the creep curves with various loading rates. Figure 8a,b summarizes the correlations between nanoindentation hardness and loading rate for Zr-Cu-Ni-Al and La-Co-Al, respectively. The indentation hardness is increased with the increasing loading rate (strain rate) for both samples, which confirms the positive strain rate sensitivity. In comparison, the growing rate of hardness on the loading rate is much smaller in La-Co-Al than in Zr-Cu-Ni-Al, i.e., hardness increases from about 3.32 GPa to 3.37 GPa for La-Co-Al (growing rate ~1.5%), and from 7.9 GPa to 8.3 GPa for Zr-Cu-Ni-Al (growing rate ~5%), as the loading rate increases from 0.035 mN/s to 0.75 mN/s. It is worth noting that the loading rate effect on hardness is consistent with that on creep flow for both samples, i.e., positive for Zr-Cu-Ni-Al and insensitive for La-Co-Al. The promoting effects of the loading rate on hardness and creep flow in Zr-Cu-Ni-Al are commonly observed in metallic glasses, which could be explained from the perspective of structure agitation and the generation of excess free volume. For the La-Co-Al film, on the other hand, such a tiny variation of hardness illustrates that the process of plastic deformation and the structure state at the peak load do not change as the loading rate increases. As a consequence, the creep behaviors of La-Co-Al would be insensitive to the variation of loading sequences. The computed values of strain rate sensitivity from steady-state creep curves are summarized in Figure 8c,d for Zr-Cu-Ni-Al and La-Co-Al, respectively. As the loading rate increases from 0.035 mN/s to 0.75 mN/s, the average value of *m* generally increases from 0.07 to 0.13 for Zr-Cu-Ni-Al whilst it lies in the range of 0.22~0.18 for La-Co-Al.

STZ volume and the atoms it contained were calculated for both samples, as listed in Figure 8e,f. For Zr-Cu-Ni-Al, STZ, the size is reduced to about 40 atoms under 0.75 mN/s and enlarged to 60 atoms under 0.035 mN/s. The results presented here could be connected to the loading rate-dependent creep behaviors. In Choi et al.’s work, it was also revealed that the STZ size of a Zr-Cu-Ni-Al-Ti metallic glass was decreased from 29 atoms to 17 atoms as the loading rate increased from 0.5 mN/s to 10 mN/s, using the nanoindentation statistical method which relies on the cumulative distribution of yield stress [52]. For La-Co-Al, on the other hand, the fluctuation of average STZ sizes with loading rate is quite small, at around 32 ± 2 atoms. Clearly, the loading rate effect on the STZ size of metallic glasses using the stress relaxation method is weak and composition-dependent. It was revealed that STZ size is closely connected to the free volume content [49], STZ size increases from 25 atoms to 33 atoms after sub-*T*_g_ annealing in a Zr-Cu-Ni-Al-Ti bulk metallic glass. As it is conceived that more excess free volume is generated beneath the indenter at a higher loading rate, a smaller STZ size can be expected at 0.75 mN/s for Zr-Cu-Ni-Al. For La-Co-Al, on the other hand, the free volume content might be insensitive to the loading rate, as mentioned above, hence the small variation in STZ size.

### 3.3. Holding Time Effect

**Figure 9:**
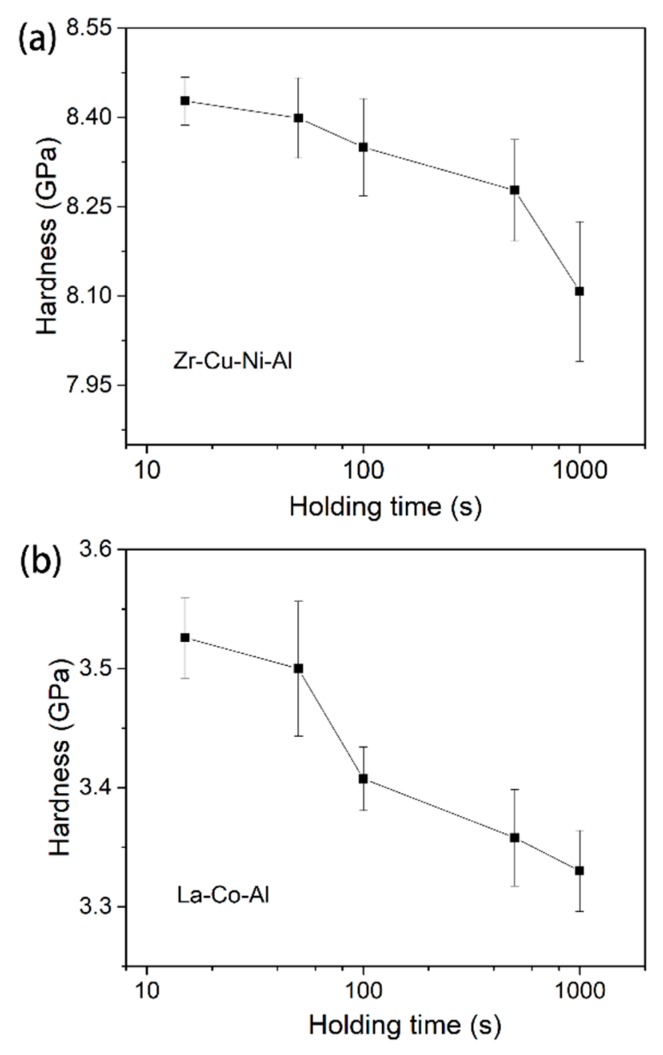
Hardness at the onset of the holding stage as a function of holding time for (**a**) Zr-Cu-Ni-Al and (**b**) La-Co-Al.

**Figure 10:**
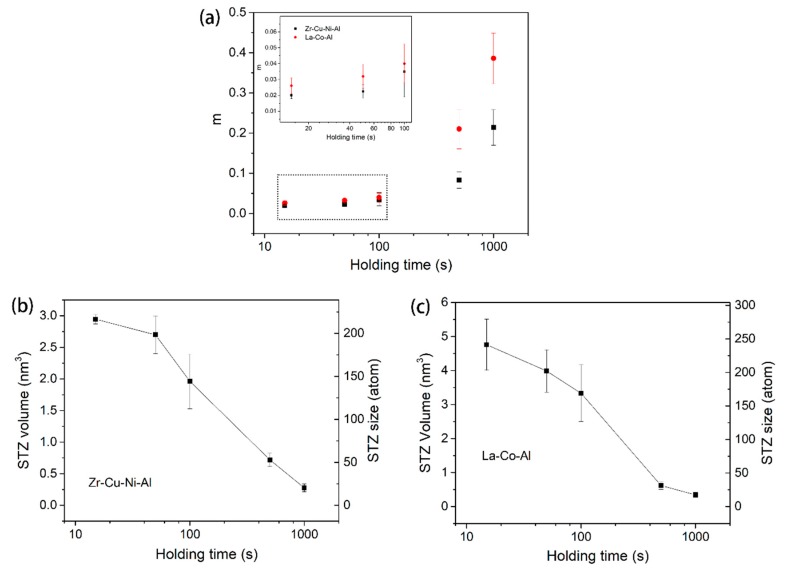
(**a**) strain rate sensitivity as a function of holding time for both samples; STZ size and volume as a function of holding time for (**b**) Zr-Cu-Ni-Al and (**c**) La-Co-Al.

As illustrated in Figure 3d, strain rate sensitivity is not a constant throughout the whole holding stage, even under fixed testing conditions. Clearly, the value of SRS *m* increases with the holding time. However, stiffness *S* is also changed with pressed depth, which is important for calculating the hardness and strain rate. Strictly speaking, we could not directly obtain the correlation between *m* and the holding time by differentiating the ln*H*-ln$\dot{\varepsilon }$ curve, in which *S* is fixed for simplicity. In order to ensure the accuracy of the holding time effect on STZ size, creep tests with various durations were performed. Figure 9 shows the hardness at the onset of the holding stage for both samples, as a function of the holding time. The decrease in hardness was due to the fact that *S* is increased with pressed depth (holding time). Figure 10a shows the correlation between strain rate sensitivity and holding time. In comparison to the result of the 500-s holding test, *m* is nearly doubled after 1000 s holding, i.e., 0.21 for Zr-Cu-Ni-Al and 0.4 for La-Co-Al. As the holding time decreases to 100 s, *m* precipitously reduces to below 0.04 for both samples, which can be more clearly recognized in the inset of Figure 10a. Then, the decrease in *m* tends to be stable, which are 0.02 and 0.025 in the 15-s holding tests for Zr-Cu-Ni-Al and La-Co-Al, respectively. The holding time effect indicates that strain rate sensitivity could significantly grow from transient creep to steady-state creep. What is more, *m* is always larger in La-Co-Al than in Zr-Cu-Cu-Ni at all events, while the *m* gap between the two samples is gradually narrowed as the holding time is shortened, and the values are very close in the 50-s and 15-s holding tests. For long-term holding, the creep flow could turn into a steady-state stage, the deformation behavior of which mainly depends on the intrinsic plastic resistance of the sample. For short-term holding, however, the apparatus error-induced fluctuation plays an important role in the recorded indenter displacement at the transient creep, such as the indenter “overshot” at the beginning of the holding stage, which weakens the creep discrepancy between La-Co-Al and Zr-Cu-Ni-Al. Thus, the variation in the *m* gap between the two samples in Figure 10a could be qualitatively explained.

The STZ volume and atoms at each holding event are calculated and summarized in Figure 10b,c. For both samples, STZ sizes shrunk from about 230 atoms to 18 atoms as the holding stage was extended from 15 s to 1000 s. This strong holding time effect on STZ size could be conceivable as being mainly due to the conspicuous enlargement of strain rate sensitivity. Being different from the mechanism of the indentation size effect on STZ size, the holding time effect described here is dominated by the extrinsic method. The different creep flow stages could be the key reason for the large change in STZ size. The apparatus error for short duration, as well as the thermal drift in long-term holding, could also be considerable for the duration-dependent STZ size.

## 4. Conclusions

In summary, nanoindentation creep measurements were conducted on as-cast Zr-Cu-Ni-Al and La-Co-Al metallic glassy films with a standard Berkovich tip. Based on the strain rate sensitivity of the creep curve, the shear transformation zone (STZ) size was estimated and its correlation with the testing conditions was revealed. The effects of indentation size, loading rate, and holding time on STZ size were systematically studied. Several conclusions could be drawn:

(1) The estimated STZ sizes using the creep method correspond well with the theoretical simulation. Under the same testing conditions, STZ size is detected to be larger in Zr-Cu-Ni-Al than in La-Co-Al, which could be due to the higher glass transition temperature (*T*_g_).
(2) The indentation size effect can be clearly observed in both samples; STZ size increases quickly with increasing pressed depth.
(3) The loading rate effect on STZ size is weak and composition-dependent. STZ size decreases slightly in Zr-Cu-Ni-Al and changes little in La-Co-Al, even though the loading rate is increased by more than an order of magnitude.
(4) Holding time is particularly important in estimating STZ size. STZ size would artificially decrease from several hundreds of atoms to no more than twenty as the holding stage increases from 15 s to 1000 s.
